# Study protocol: a randomized controlled trial testing the effectiveness of an online mindset intervention in adolescents with intellectual disabilities

**DOI:** 10.1186/s12888-018-1939-9

**Published:** 2018-12-04

**Authors:** Fenneke L. M. Verberg, Petra Helmond, Geertjan Overbeek

**Affiliations:** 1 Pluryn Research & Development, P.O. Box 53, 6500 AB Nijmegen, The Netherlands; 2 Research Institute of Child Development and Education, Faculty of Social and Behavioural Sciences, University of Amsterdam, P.O. Box 15776, 1001 NG Amsterdam, The Netherlands

**Keywords:** Intellectual disabilities, Mindset, Implicit theories, Empowerment, Online intervention, Adolescents

## Abstract

**Background:**

Adolescents with intellectual disabilities have an increased risk of developing academic, social, and psychological problems compared with non-disabled peers. These difficulties might have an impact on the implicit theories—or so called mindset—of these youth. Youth with a fixed mindset believe that their attributes are static while youth with a growth mindset believe their attributes are malleable. A growth mindset can positively affect the academic and psychosocial development of youth and can be stimulated by so called ‘mindset interventions’. Nevertheless, mindset interventions specifically adapted to adolescents with intellectual disabilities are non-existing.

**Methods/design:**

The aim of the present study is to conduct a randomized controlled trial (RCT) to test the effectiveness of the online mindset intervention “The Growth Factory” aimed to teach adolescents with intellectual disabilities a growth mindset and thereby positively impacting their psychosocial development. The RCT targets adolescents (12–23 years) with mild to borderline intellectual disabilities (IQ 50–85) admitted to residential care or special education. Participants will be individually randomized to the intervention (*n* = 60) or control (*n* = 60) group. The intervention group will individually participate in the six sessions of “The Growth Factory” and the control group will receive care as usual. Primary outcome will be mindset. Empowerment, behavior problems, self-esteem, treatment motivation, therapeutic alliance, challenge seeking, and the impact of social exclusion will be included as secondary outcome measures. Moreover, moderation (i.e., intervention satisfaction, IQ, age, baseline mindset, gender) and mediation effects will be investigated. Self-reported and mentor assessments will be administrated at baseline, post-test and at three (except mentor assessment) and six months follow-up.

**Discussion:**

This paper describes the design of a RCT examining the effectiveness of the online mindset intervention “The Growth Factory” aimed to empower adolescents with intellectual disabilities. If effective, “The Growth Factory” makes an important contribution to the treatment and psychosocial development of adolescents with intellectual disabilities in residential care and special education. Due to the online approach, implementation will be efficient and cost-effective and therefore the intervention “The Growth Factory” can be used on large scale.

**Trial registration:**

Dutch Trial Register NTR5460. Registered 2 October 2015.

## Background

Youth with intellectual disabilities are highly vulnerable and experience more difficulties and delays in academic, social, and adaptive skills [1–4]. In addition, research shows that young people with intellectual disabilities have an increased risk of developing emotional and behavioral problems [2–9]. Youth with intellectual disabilities show more externalizing problems, such as attention problems and aggressive behavior, than their non-disabled peers [2, 5–7]. The same holds for internalizing problems, such as depression and anxiety [2, 3, 5–7, 10, 11]. Moreover, youth with intellectual disabilities often suffer from overprotective care [12–14]. Due to their disabilities, many youth with intellectual disabilities are restricted by caregivers’ low expectations and fear for their safety [14]. Overprotective care may hamper identity building, independence and autonomy in youth with intellectual disabilities, and is related to psychosocial maladjustments [12–14].

The experience of academic and psychosocial problems, amongst other factors, might have an impact on the implicit theories of youth with intellectual disabilities. Implicit theories—also referred to as mindset—are core assumptions about the malleability and controllability of particular attributes such as intelligence, emotion, behavior, and personality [15, 16]. These implicit theories create a framework for interpreting the meaning of events in one’s world. Two types of mindsets can be distinguished, that is a fixed mindset (an entity view) and a growth mindset (an incremental view).

In particular, youth with a fixed mindset consider attributes such as intelligence and personality to be static and unchangeable. For example, they might believe that people have certain personality traits that cannot be altered [15]. For this reason, effort is seen as useless and hard work will be without results or success. Furthermore, youth with a fixed mindset will tend to avoid challenging situations and will see setbacks as threatening and self-defining because it indicates a general lack of ability [15, 16]. As a result, people endorsing a fixed mindset may not achieve their full potential. In contrast, people with a growth mindset believe people’s characteristics have the potential to change and see these attributes to be dynamic. For example, they may believe that everyone can take steps to develop their personality and behavior over time [15]. For this reason, those who believe these attributes are malleable tend to engage in behaviors that will help them to develop their abilities, such as expanding effort to improve and embrace challenges as opportunities to grow [15, 16]. As a result, youth with a growth mindset intend to use their full potential and therefore might reach higher levels of academic achievement and psychosocial functioning [16–18]. The present study focuses specifically on the impact of a growth mindset on enhancing the psychosocial development of youth with intellectual disabilities.

Indeed, an extensive amount of research has shown significant associations between mindsets and a wide range of psychological outcomes [17–23]. The psychological constructs in the present study to assess effectiveness outcomes of the intervention will be discussed. Research has shown that a growth mindset is associated with psychological empowerment [19]. Empowerment is the experienced personal competence and perceived control to handle important matters [24–26]. In addition, the belief in the malleability of one’s own capabilities impacts one’s self-regulation of behavior and motivation [17, 19, 26–28]. For example, people with a growth mindset set goals focused on learning to increase their ability [17, 27, 28] as they are more likely to prefer challenging activities compared to people with a fixed mindset [20]. Furthermore, people with a growth mindset employ mastery-oriented strategies by displaying more willingness to work hard and persistently, even when faced with adversity, to reach their goals [17, 28–30]. Consequently, people endorsing a growth mindset are more likely to be confident in successfully making a change and therefore more likely to be motivated for treatment (i.e., treatment readiness) to improve their emotions and behavior [21, 29]. Subsequently, a growth mindset might also be related to building positive therapeutic relationships [22, 30, 31]. For example, people who have a growth mindset believe in personal responsibility for working hard and achieving progress and therefore are more likely to evaluate their relationship with their counselor as collaborative and productive than people with a fixed mindset [30]. In addition, a growth mindset is related to lower levels of internalizing and externalizing problems, such as anxiety, depression, and aggressive behavior [18, 32–34]. Moreover, mindsets are associated with (long-term change in) self-esteem, with people with a fixed mindset showing lower levels of self-esteem and a downward spiral in self-esteem levels in response to new (academic) challenges compared to those with a growth mindset [17, 34]. Finally, mindsets are related to people’s social relationships [23, 35–38]. Specifically, a fixed mindset has been related to a greater desire for vengeance when adolescents recalled recent conflicts in their lives [23]. Moreover, children with a fixed mindset are more likely to demonstrate internalizing and externalizing health problems when victimized [37, 38].

In sum, a growth mindset can positively impact adolescents’ academic, social, and psychological development. Therefore, so called ‘mindset interventions’ have been developed to teach children and adolescents a growth mindset. Mindset interventions are brief psychological interventions based upon the previously described scientific research concerning implicit theories of intelligence and personality [39]. A key message of mindset interventions is that attributes are malleable and can be changed. Thus, these interventions show the plasticity of the brain and the impact of effort and practice. Furthermore, the focus of these mindset interventions is on implicit and unconscious beliefs instead of teaching new skills or behavior [39]. Mindset interventions are generally one to eight sessions long and are executed face-to-face or using a computer program using an individual or group format.

Interestingly, mindset interventions have been shown to be successful in stimulating a growth mindset and subsequently positively impacting adolescents’ academic performance and psychosocial functioning [27, 37, 39–43]. First, mindset interventions showed the predicted effect of increasing a growth mindset in adolescents [19, 37, 41, 44, 45]. Second, mindset interventions significantly increased feelings of empowerment in youth [19]. Third, previous research found that after a short growth mindset manipulation youth were more willing to take on challenges compared to youth who received a fixed manipulation [20, 43]. Fourth, providing youth with conduct problem and psychopathic features with an intervention including a growth mindset component demonstrated increased positive emotion and improvement in treatment amenability (i.e., awareness of problems, motivation to change, and consideration and tolerance of others) [42]. Fifth, mindset interventions can make an important contribution to the prevention and reduction of behavioral problems [41, 44]. In particular, a brief mindset intervention teaching adolescents that people can change prevented internalizing problems (e.g., symptoms of depression) [41] and externalizing problems (e.g., aggression) [39]. Sixth, a single session intervention teaching a growth mindset of personality was effective in preventing a decline in self-esteem [41]. Finally, mindset interventions had a positive impact on social relationships [23, 39]. For example, youth who have participated in a mindset intervention responded less aggressive and more prosocial in reaction to social rejection compared to youth in the control group [39].

Despite these impressive findings, previous mindset research has been mainly conducted in educational settings with adolescents without disabilities. However, according to a few studies, children and adolescents with intellectual disabilities are more likely to endorse a fixed mindset than peers without disabilities [43, 46, 47]. Furthermore, research shows that a growth mindset is related to higher levels of empowerment and self-esteem in youth with intellectual disabilities [46]. Also, higher levels of a growth mindset are related to lower levels of internalizing problems, attention problems, externalizing problems, and total behavior problems [46] and are not associated with challenge avoidance [43, 47]. These results suggest that teaching a growth mindset might make a significant contribution to the development of youth with intellectual disabilities. However, mindset interventions adapted to the needs of adolescents with intellectual disabilities are lacking. Therefore, we developed a brief six session online mindset intervention “The Growth Factory” aimed to teach youth with intellectual disabilities a growth mindset.

In a randomized controlled pilot study (*n* = 59) we showed that it was feasible to implement the online intervention in practice and that the majority of adolescents with psychiatric problems often combined with intellectual disabilities evaluated “The Growth Factory 1.0” positively [48]. The pilot study also demonstrated that the intervention significantly increased a growth mindset and feelings of empowerment of adolescents with intellectual disabilities and/or psychiatric problems in comparison with a control group—although the intervention did not show the expected beneficial downstream effects on internalizing problems, externalizing problems, and self-esteem. Based on these findings, and on participant and trainer evaluations in the pilot study, “The Growth Factory 1.0” was further improved into “The Growth Factory 2.0”—in this article further referred to as “The Growth Factory”—to increase the effectiveness of the intervention for adolescents with intellectual disabilities. Some important changes were the correction of technical errors, the addition of a participant workbook, and the addition of two assignments on the relationship between cognitions and behavior.

The primary aim of the present study therefore is to examine the effectiveness of the online intervention “The Growth Factory” using a full scale randomized controlled trial (RCT) with four measurement moments. “The Growth Factory” aims to empower youth with intellectual disabilities by stimulating the development of a growth mindset and thereby positively impacting their psychosocial development. We hypothesize that adolescents in the intervention group will show larger increases in growth mindset (primary outcome). Furthermore, we hypothesize that adolescents in the intervention group will show greater improvements in empowerment, self-esteem, treatment motivation, and therapeutic alliance as well as a larger reduction of internalizing problems, attention problems, externalizing problems, and total behavior problems compared with adolescents in the control group (secondary outcomes). Finally, we hypothesize that adolescents in the intervention group will seek challenges more and will be less negatively impacted by social exclusion compared with adolescents in the control group (secondary outcomes). In addition, the secondary aim of this study is to gain insight for whom the intervention “The Growth Factory” is effective (i.e., moderation) and how the intervention works (i.e., mediation). Intervention satisfaction, level of intellectual disability (mild intellectual disability versus borderline intellectual functioning), and baseline mindset will be tested as moderators of the effects of the online mindset intervention. We hypothesize that adolescents with higher intervention satisfaction scores, borderline intellectual functioning, and a fixed mindset at baseline will show larger increases in growth mindset compared to adolescents with mild intellectual disabilities, who report less satisfaction with the intervention, and a more growth oriented mindset at baseline. In addition, we will explore whether age and gender moderate the intervention effect. Finally, we will test the mediating role of mindset on the effect of the mindset intervention on the secondary outcomes measures.

## Methods

The study design will be reported in accordance with the CONSORT 2010 statement for reporting parallel group randomized trials [49]. The Ethics Committee of the University of Amsterdam in the Netherlands has approved the study (2015-CDE-4518). Moreover, the study is registered in the Dutch Trial Register for RCT’s (NTR5460).

### Design

**Fig. 1:**
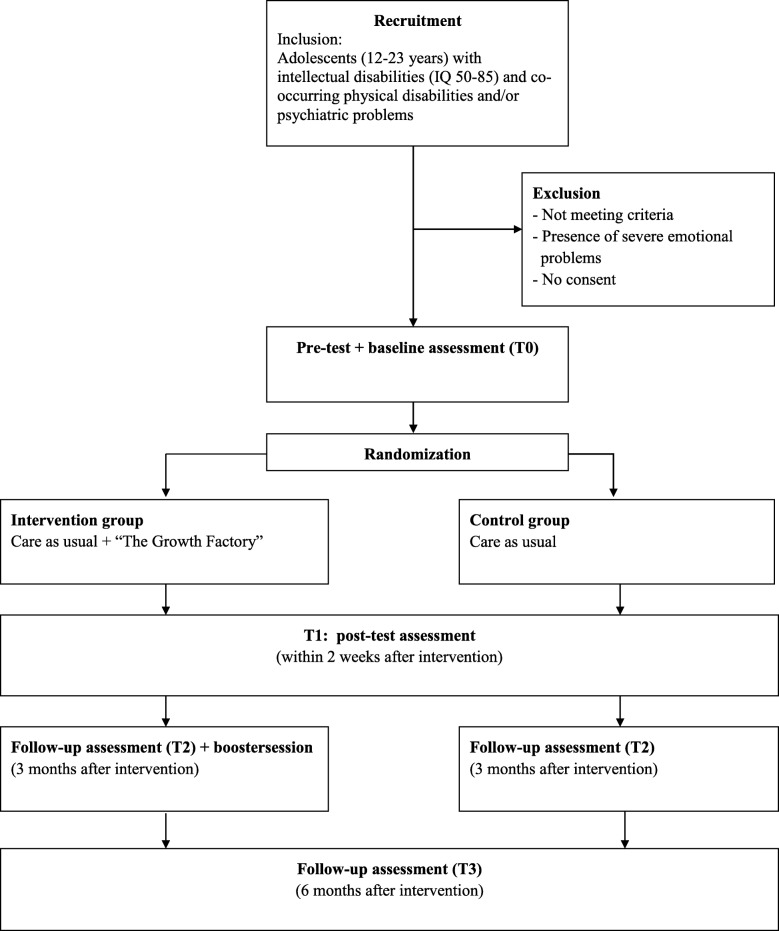
Study design. Flow diagram of recruitment, inclusion and exclusion criteria, randomization and different assessments

The present study involves a randomized controlled trial with two conditions: an intervention group and a control group with four measurements at pre-test, post-test, follow-up at 3 months and 6 months. Figure 1 shows a schematic overview of the design in the present study.

### Participants

Participants that will be selected for the study are (late) adolescents (12 to 23 years) with mild to borderline intellectual disabilities, including youth with mild intellectual disabilities (IQ 50–69) and borderline intellectual functioning (IQ 70–85) and deficits in adaptive functioning. Besides an intellectual disability participants could have accompanying physical disabilities or psychiatric problems. Participants are clients in residential care or students in special education. Exclusion criteria are the presence of severe emotional problems hindering participation in the study, such as extreme aggression problems or an acute unstable mental condition. Participants who agree to participate will be included in the study when both adolescent and parents or legal representative provide written informed consent.

A power analysis [50] was performed to calculate the sample size required in the present study. Based on previous research on the effectiveness of mindset interventions in education [39], the expectation is to find a small to medium effect (*d* = 0.25). The power calculation (two-tailed, alpha 0.05, statistic power 0.80) based on a three measurements design shows that 106 participants are necessary. Therefore, our aim is to include *N* = 120 participants (*n* = 60 intervention group; *n* = 60 control group) in the RCT taking into consideration 10% attrition.

### Procedure

Treatment coordinators of the institute and the school psychologists will screen youth for inclusion and exclusion criteria. After that, parents or legal representatives will receive an information letter containing a digital link and response letter to sign-up if they wish their child to participate in the study (active informed consent). In addition, adolescents will be informed approximately a week before the first screening by two research assistants in their classroom or group. If potential participants are absent or if they need extra information, information will be given individually. If adolescents also agree with participation, active written informed consent will be obtained. After that, participants will be randomly allocated to either intervention group or control group. Randomization will take place at individual level using a stratified block design to ensure equality between conditions. The stratified block randomization will be based on three factors: gender, age, and IQ. Parents, mentors, and teachers will be informed by a letter about the condition their child is assigned to.

Before filling out the pre-test, all participants will complete a short questionnaire containing questions regarding their living group or class and additional therapies (i.e., physiotherapy, speech therapy, social skills training). After that, the pre-test (T0) will be assessed. After the pre-test, youth in the intervention group will participate in the intervention “The Growth Factory” with six sessions lasting 25 to 40 min. Moreover, participants will receive care as usual parallel with “The Growth Factory”. Parents will also receive a login to be able to follow the sessions at home. Youth in the control group will receive care as usual. After completion of the trial participants in the control group will be given the opportunity to participate in “The Growth Factory”.

After completing the sessions of “The Growth Factory”, all participants will be assessed at post-test (T1) and a follow-up at 3 months (T2) and 6 months (T3). Furthermore, participants in the intervention group will also receive a booster session directly after the 3 months follow-up. Research assistants will guide the participants individually during the assessments and intervention in a silent room using a protocol. In line with the assessment protocol all questions will be read aloud and a standardized clarification for questions will be used. Additionally, assistance will be provided in case participants need help to complete the forms. To minimize any connection between the intervention and the measures, different research assistants will collect the measures apart from the one who guided the youth during the sessions. All research assistants will participate in four training sessions and have a bachelor or master degree or are in the final year obtaining their degree.

During the sessions, participants in the intervention group will receive a small gift (i.e., a refrigerator magnet with the five steps to ask for help in an appropriate way, and a bracelet with the ‘recipe of growth’) after completing session five and six. Furthermore, all participants receive a thumbs-up flashlight and small ‘brain stressball’ after completing the first and second follow-up measurement.

### Intervention

“The Growth Factory” is an online intervention that aims to empower adolescents with intellectual disabilities. The intervention is based on scientific research on implicit self-theories and mindset interventions by Carol Dweck and David Yeager [16, 36, 39, 51, 52]. A multidisciplinary team of professionals and youth has developed the intervention using a Dutch guideline for effective interventions for people with intellectual disabilities [53]. By using an online approach in the intervention we were able to address the information processing needs of youth with intellectual disabilities. For example, by providing visual and auditory support, using interactive assignments and animations, and the possibility to repeat parts of the session. The adapted intervention has been tested in a pilot study with adolescents with intellectual disabilities and/or psychiatric problems [48]. After that, improvements were made which resulted in the current program “The Growth Factory (2.0)” [54].

“The Growth Factory” consists of six sessions and one booster session, each lasting for 25–40 min. Youth participate in the sessions in a silent room under guidance of a research assistant using a protocol. The assistant will check whether the participant understands the information and provide help if needed. Furthermore, during the sessions the assistant will make observations concerning understanding, pauses needed, attitude, and attention. These and any other important observations will be written down on a checklist after each session.

Before the start of the first session, participants choose an avatar who will guide them through the sessions. Each session has the same structure: (1) previous week’s homework assignments are discussed with the research assistant, (2) a welcome by the avatar including a summary of the previous session and an introduction of the upcoming theme, (3) an animation clip in which the content of the session is explained, (4) two interactive assignments, (5) a summary with the most important messages of the session, (6) a goodbye by the avatar, (7) participants rate their satisfaction with the session, and (8) homework assignments are explained by the research assistant.

In the first session, the participants learn about the plasticity of the brain, that the brain is more like a muscle and that people can grow their brain by ‘exercising’. Specifically, youth are told that the connections in their brain multiply and get stronger when they use them. In session two, participants learn about growth and fixed mindsets. Specifically, they learn that people with a growth mindset believe they can develop their abilities through (mental) exercise. People with a fixed mindset believe people cannot really change and are convinced that abilities, cognitions, and personality are set. In session three, participants learn that a growth mindset helps to accomplish goals. Moreover, they learn that people with a growth mindset will embrace challenges, persist in the face of setbacks, and see effort as a strategy needed to reach one’s potential. Also, participants practise with so called ‘grow thoughts’. In session four, participants learn about the ‘recipe of growth’, which consist of three important ingredients. The first two ingredients of the recipe for growing your brain: ‘effort’ and ‘good strategies’ are taught in the fourth session. To develop abilities and skills, both effort and practise are of great importance. Furthermore, finding the best strategy to accomplish a goal is important. In the fifth session, the third ingredient ‘help from others’ is explained. Participants learn that sometimes it can be rewarding to ask for help or accept help from others. They also learn the five steps to ask for help in an appropriate way. The sixth session is a compilation of the previous sessions. The most important information is repeated. Moreover, this session will be used as the booster session after the 3 months follow-up. In addition, in sessions three, four, and five movie clips are shown about ‘peer role models’ in which these peers share their experiences in how a growth mindset helped them in encountering problems and accomplishing goals. Also, these three sessions contain additional assignments at the end of the session to practise the content of the session with the research assistant. Session three and four contain exercises based on the principles of cognitive behavioral therapy. Youth practise to recognize negative thoughts in a social situation and change these thoughts into so called ‘growth thoughts’. In session five youth practise asking for help using the five steps in a role play.

Every week, the participants receive two messages by mobile phone and/or email containing a reminder of the session’s content or a short assignment. The purpose of these messages is to improve the transfer from the online intervention into daily life. Furthermore, each participant receives a workbook. Every week, a few homework assignments need to be completed. The research assistant discusses homework assignments with the participant before starting the next session.

### Care as usual

Participants assigned to the control condition will receive care as usual. Adolescents recruited from a special education school attend the school curriculum and are supervised by a mentor teacher. In addition, a school psychologist is involved in the educational learning process and provides specific orthopedagogical advise. Each student receives a ‘developmental perspective plan’ based on the youth’s specific developmental needs. Furthermore, additional therapies are offered depending on the youth’s care need, such as resilience training and creative arts therapies (e.g., art, music, and/or dramatherapy). Specifically, for youth with physical disabilities physiotherapy, ergotherapy, and medical assistance are offered. Adolescents recruited from a specialized residential care institute receive an ‘individual treatment plan’ in which treatment goals and plans are formulated based on the youth’s specific developmental needs. A multidisciplinary team is involved in these treatment programs. One of the group care workers is the youth’s mentor who provides guidance and support based on the treatment goals and plans. Furthermore, additional therapies are offered, such as physiotherapy, medication management, and resilience training; and sometimes a family social worker is involved. Also, in residential care youth receive medical assistance when needed. Youth care workers provide care using a “strength based approach” which focusses on the individuals’ strengths, potential, and self-determination.

### Instruments/measures

Instruments will be adjusted to reduce the complexity of the questionnaires for the participants with intellectual disabilities using the Dutch guideline for the development and adjustment of diagnostic instruments for people with intellectual disabilities [55]. The answering categories of the different questionnaires will be unified into one format of answering catergories ranging from ‘completelyuntrue’ to ‘completely true’, and coloured emoticons corresponding with the answering categories will be added. Difficult words and sentences will be simplified or slightly rephrased to avoid misunderstandings due to literal interpretation. The pilot studies showed the questionnaires to be suitable for youth with intellectual disabilities [46]. Table 1 shows an overview of study outcome measures and the informants that will be involved in each assessment.

**Table 1:** Overview of assessments

	T0	Session 1–6	T1	T2	T3
Adolescent					
Mindset (MQ)	x		x	x	x
Empowerment (EMPO Youth 2.0)	x		x	x	x
Behavior problems (BPM-Y)	x		x	x	x
Self-esteem (RSES)	x		x	x	x
Treatment motivation (MYTS)	x		x	x	x
Therapeutic alliance (TASC-r)	x		x	x	x
Challenge seeking (Puzzles)	x		x	x	x
Impact of social exclusion (Cyberball game)	x		x	x	x
Intervention satisfaction (SRS)		x			
Mentor^a^					
Mindset (MQ)	x			x	x
Empowerment (EMPO Youth 2.0)	x			x	x
Behavior problems (BPM-P)	x			x	x

^a^Mentor is defined as school mentor or social worker from the group

### Screening measures

For all participants, information regarding gender, age, IQ scores, and diagnosis will be provided by the treatment coordinator or school psychologist. Participants will provide information about setting (residential care group or homestay) and attributional treatment (e.g., physiotherapy, speech therapy, social skills training).

### Primary outcome measure

*Mindset* will be measured with the Mindset Questionnaire (MQ) [46]. The MQ consists of two parts: (1) two subscales measuring youth’s implicit theories: mindset emotion/behavior (6 items, e.g., ‘I can control the feelings I have’) and mindset intelligence (3 items, e.g., ‘I can learn new things, but I can’t really change my basic intelligence’), and (2) the subscale ‘perseverance’ measuring youth’s self-regulatory behavior (9 items, e.g., ‘If something does not work, I quit’ and ‘By practising a lot I’m getting better’). The original 6-point Likert scale was replaced by a 5-point Likert scale [56], ranging from *1* (‘completely untrue’) to *5* (‘completely true’), with higher scores indicating a higher endorsement of a growth mindset. Furthermore, the pilot studies showed that the MQ is suitable for youth with intellectual disabilities [46] and the validity and reliabilities of the subscales ranged from just sufficient to satisfactory [46]. In addition to self-report of youth, participants mentor will complete the MQ about their view on participant’s mindset and self-regulatory behavior.

### Secondary outcome measures

*Empowerment* will be measured with the Dutch questionnaire ‘EMPO Jongeren 2.0’ (EMPO Youth 2.0) [25]. The EMPO Youth 2.0 consists of 16 items measured on a 5-point scale ranging from *1* (‘completely untrue’) to *5* (‘completely true’). The subscale ‘intrapersonal’ contains 9 items (e.g., ‘I am in control of myself’) as the scale ‘interactional’ consists of 7 items (e.g., ‘I make use of advice or support from people around me, if necessary’). The sum of the scores on all items yields a total empowerment score. The EMPO Youth 2.0 demonstrates sufficient reliability [25, 46]. Also adolescent’s mentor will complete the EMPO Youth 2.0 questionnaire about their view on adolescent’s empowerment.

*Internalizing, attention and externalizing behavior problems* will be assessed using the Dutch translation of the ‘Brief Problem Monitor-Youth’ (BPM-Y) [57, 58]. The BPM-Y contains 19 items measuring internalizing problems (6 items), attention problems (6 items), and externalizing problems (7 items). The sum of the scores on all items yields a total problems score. The items will be rated on a 3-point scale. The original categories ‘not true’, ‘somewhat true’, and ‘very true’ were replaced with ‘completely untrue’, ‘not true/ not untrue’, and ‘completely true’ to match with the answering categories in the other questionnaires. An example item of the externalizing problems scale ‘I threat other people’. The BPM-Y demonstrates sufficient and satisfactory reliability [46, 57]. In addition, adolescent’s mentor will complete the questionnaire about their view on behavior problems using the parent version of the BPM, the ‘Brief Problem Monitor-Parent form’ (BPM-P) [57]. This scale offers good to excellent internal consistency and acceptable to good test-retest reliability [57].

*Self-esteem* will be assessed using the Dutch translation of the ‘Rosenberg Self-Esteem Scale’ (RSES) [59]. The scale is a 10-item Likert scale with items answered on a 4-point scale. The original four answering categories ‘strongly agree’ to ‘strongly disagree’ were reformulated into ‘completely untrue’ to ‘completely true’ to match the answering categories in the other questionnaires. An example item is ‘I am able to do things as well as most other people’. The instrument possesses satisfactory reliability [46, 60].

*Treatment Motivation* will be measured with the Motivation for Youth’s Treatment Scale (MYTS) [61], an eight item measure that assesses problem recognition (e.g., ‘My behavior is making my life worse’) and treatment readiness (e.g., ‘If I attend counseling I think my life will get better’). In addition, completion of the MYTS yields a total score for overall motivation. The original 5-point answering categories ‘strongly disagree’ to ‘strongly agree’ were replaced with ‘completely untrue’ to ‘completely true’. Three changes were made in the treatment readiness scale, because participants at the institutional care already receive counseling. Therefore, the word ‘extra’ was added to the sentences about getting counseling (e.g., ‘Getting *extra* counseling seems like a good idea to me’). Furthermore, the sentence ‘Complete these two questions only if you get extra counseling’ was added because not all pupils already receive extra assistance. Moreover, the question ‘I get extra counseling because others think I need it’ was added to complete the questionnaire. The original version of the MYTS offers a brief and reliable tool to assess treatment motivation among youth and caregivers. The internal consistency of this instrument is good [61].

*Therapeutic alliance* will be measured with the Dutch translation of the Therapeutic Alliance Scale for Children, revised (TASC-r) [22]. The instrument contains 12 items covering both postive and negative aspects of therapeutic alliance, and distinguishes between the affective bond (6 items, e.g., ‘I like spending time with my therapist’) and client-therapist collaboration (6 items, e.g., ‘I’d rather do other things than meet with my therapist’). In addition, completion of the TASC-r yields a total score for overall alliance. The word ‘therapist’ is reformulated into ‘mentor’ to better fit the target group. Before participants start to complete the questionnaire the assistant will make clear about whom (mentor from school or youth care group) the questionnaire has to be filled in. The original items are rated on a 4-point Likert scale ‘not true’ to ‘very much true’. These categories were changed into the 5-point answering categories ‘completely untrue’ to ‘completely true’ to match the answering categories of the other measures used. The scale has demonstrated adequate internal consistency and validity in previous investigations [22, 62].

*Challenge seeking* will be assessed by using the experimental task ‘puzzles’, based on the measure ‘challenge-seeking: hypothetical scenario’ [20] and the idea of Koestner’s ‘hidden figure puzzles’ [43] to measure willingness to seek challenges. The task consists of three puzzles, each presented in an envelope. The envelops contain respectively an ‘easy’, ‘medium’, and ‘difficult’ puzzle. Participants will be asked to choose a puzzle and answer the question why that specific puzzle is chosen, but participants do not actually complete the puzzle. The choice of challenge level and the reason why that specific puzzle is chosen will be reported by the research assistant.

*Impact of social exclusion* will be measured by using the ‘Cyberball game’ [63]. In this game, the participant plays two virtual ball-toss games with three others who are presented to be real and connected through a network. The ‘others’ are in fact controlled by the computer program. In the first game the youth plays a ‘normal’ tossing ball game and will receive the ball as often as the other players. In the second game it is tested whether the participant will be affected by victimization. After receiving the ball a few times the participant is excluded by the other players and no longer receives the ball during the game. After completing both games, a post experimental questionnaire [64] will be assessed. The questionnaire contains 11 statements to measure the impact of the exclusion on belonging (‘I feel I belonged to the group’), mood (e.g., ‘How do you feel?’), perception of group cohesiveness (e.g., ‘I do not like the other players’) and intensity of ostracism (e.g., ‘I feel I was being excluded by the other players’). Three questions about meaningful existence, control, and self-esteem of the original 12 items questionnaire were deleted. Two new statements served as a manipulation check (e.g., ‘The other players are real participants’). The original 9-point answering categories were replaced by a 5-point scale with different categories, e.g., ‘completely untrue’ to ‘completely true’, ‘very bad’ to ‘very good’, and ‘very sad’ to ‘very happy’. Furthermore, the desire for vengeance after exclusion is measured by providing participants the opportunity to take revenge by allocating hot sauce to one of the peers by whom they were excluded during the second Cyberball game. Participants will be told that the other player dislikes spicy food, but has to consume the entire amount of hot sauce anyway. The research assistant will report the amount of allocated hot sauce in grams using a digital weighing scale. Finally, the youth will play the first version of the Cyberball game again.

Participants’ safety during data collection for the Cyberball and Hot Sauce paradigms is guaranteed. First, during the experiment the exclusion experience is brief, mild, and quickly followed by an inclusion experience. Second, participants will be debriefed immediately after the experiment, following a standardized protocol. Participants will be told the exclusion happened due to a computer error and the players will not have to eat the hot sauce. Furthermore, studies in other special needs populations also obtained ethical permission for this experiment [65, 66] and have shown participants did not express regret or distress at having taken part in the Cyberball game [64, 65]. After debriefing, the participant will have to answer three more questions about their mood to check if they feel relaxed and unthreatened. The Cyberball task takes approximately 10 min.

#### Intervention satisfaction

Participants in the intervention group will grade their satisfaction after each session of “The Growth Factory” with a score from *1* (very low) to *10* (very high). This grading system is in line with the Dutch educational system. The mean of the grades per session will provide a mean session satisfaction grade. Furthermore, the mean of these satisfaction grade scores of the six sessions will be taken to construct an overall mean intervention satisfaction grade. In addition, intervention satisfaction will be measured with a Dutch translation and adaptation of the Session Rating Scale (SRS) [67]. Participants will complete four statements, e.g., ‘The assistant listened to me today’ (relationship), ‘What we did today is important to me’ (goals and topics), ‘I liked what we did today’ (approach and method) and ‘I hope next time we will do something similar’ (overall). The original 10-cm visual analog scale will be replaced by a 5-point Likert scale ranging from ‘completely untrue’ to ‘completely true’. The average score of the four items will be taken as an indicator of the satisfaction with each session (session SRS). Subsequently, to construct an overall intervention satisfaction score (intervention SRS) the average will be taken of the session satisfaction scores of the six sessions. The scale has demonstrated a satisfactory reliability and validity in previous research [67]. Finally, participants will be asked to answer two open questions as a qualitative measure of session satisfaction: ‘What did you like about today’s session?’ and ‘What did you not like about today’s session?’

### Statistical analyses

Following the intention-to-treat principle, the data from all participants randomized to either the intervention or control group will be analysed. Multiple imputations will be used for missing values at post-intervention and follow-up measurements. In addition, a completers only analysis will be conducted (i.e., participants that completed five or six sessions). The results will be reported in accordance with the CONSORT Statement [49].

Possible baseline differences between the two groups in background variables (e.g., age, gender) and relevant study variables will be examined using independent-sample t-tests. In case of differences at baseline, variables will be included as covariates in all models testing the effectiveness of the intervention. Furthermore, the effectiveness of the intervention will be analysed using repeated measures ANOVA for differences within subjects (i.e., pre-test, post-test, follow-up 1, and follow-up 2 measurements) and between subjects (intervention versus control group). In addition, intervention satisfaction, level of intellectual disability, age, baseline mindset, and gender will be tested as moderators of the effects of the online mindset intervention.

These moderator effects will be tested using three-way interactions in our repeated measures design. The effect of the mindset intervention on the secondary outcomes measures, (i.e., empowerment, internalizing problems, attention problems, externalizing problems, and total behavior problems, self-esteem, treatment motivation, therapeutic alliance, challenge seeking, and impact of social exclusion) might be mediated by mindset. This will be tested in several mediation analyses in Mplus [68].

## Discussion

The present study protocol presents a randomized controlled trial testing the effectiveness of the online mindset intervention “The Growth Factory”. The intervention aims to develop a growth mindset in adolescents with intellectual disabilities. A growth mindset leads to higher levels of academic achievement and psychosocial development [16–18]. Therefore, we expect that adolescents in the intervention group will show larger improvements in their psychosocial development compared with adolescents in the control group. The primary aim of the present study will be to investigate whether “The Growth Factory” affects the following outcomes: mindset, empowerment, behavior problems, self-esteem, treatment motivation, therapeutic alliance, challenge seeking, and impact of social exclusion. The secondary aim will be to examine which factors moderate or mediate the effect of the online intervention “The Growth Factory”.

### Strengths and limitations

To our knowledge, this is the first full scale RCT study evaluating an online mindset intervention developed for adolescents with intellectual disabilities. RCT studies are considered the gold standard for evaluating efficacy in clinical research [69]. In addition, in contrast to the most RCT studies, we will not only focus on the effectiveness of the program, but also on the moderating and mediating factors of change (i.e., for whom and how the intervention works). Furthermore, the intervention is based on previous effective mindset interventions [20, 52] and the core principles of these interventions will remain intact in “The Growth Factory”. Moreover, the intervention is specifically adapted for adolescents with intellectual disabilities using the guidelines for effective interventions for people with intellectual disabilities [53]. An additional strength of the study is that the intervention paradigm has been pre-tested in a pilot study to improve the intervention for the target group and to ensure that it fits the information processing needs of these youth with special learning needs. Another strength of this study is that different locations of the residential care institute will participate in this study, as will different schools for special education across the country. For this reason, the participants in the current study will represent the diverse population of youth with intellectual disabilities. Furthermore, the triangulation of different data sources (self- and teacher reports, behavioral tasks at the last follow-up) across four measurement moments (pre-test, post-test and a 3 and 6 months follow-up) is a strength.

However, this study also has some limitations. The first is the lack of an additional program specifically developed for mentors and parents as the environment plays a crucial role in facilitating or inhibiting the development of a growth mindset [70, 71]. To diminish this limitation parents were provided with personal login codes to be able to participate in the intervention “The Growth Factory”. However, mentors were not provided with a login code to prevent intervention contamination to the control group, because it could be possible that youth from the intervention and control group were in their class or group. Another limitation is that we will not be able to include a third condition that acts like an active control group to ensure the effects can be uniquely ascribed to the intervention “The Growth Factory”. However, previous research showed that a mindset intervention was more effective than both a passive (no intervention) as well as an active control group [40]. An additional limitation is that the present study, in contrast to many previous studies on mindset interventions, does not measure the impact of “The Growth Factory” on academic achievement. The reason for this is that standardized testing is exceedingly complex in this context—it is not always the standard in special education and varies widely across special education schools. Finally, only short- and medium-term effects (3 and 6 months follow-up) will be investigated. In this way, no conclusions can be drawn about the longer-term effects of “The Growth Factory” on the psychosocial development of youth with intellectual disabilities.

### Implications for practice

If “The Growth Factory” proves to be effective, a significant contribution to the evidence-based treatment of empowerment in adolescents with intellectual disabilities will be provided. When adolescents with intellectual disabilities believe in the malleability of their capabilities and therefore experience more control over their own lives, this will subsequently help to improve their psychosocial outcomes. Furthermore, due to the online approach, dissemination and implementation of the intervention will be efficient and cost-effective and therefore the intervention “The Growth Factory” will be able to be used on large scale in residential care institutes and at special schools.

## Conclusion

Adolescents with intellectual disabilities are more likely to endorse a fixed mindset compared to their non-disabled peers. Mindset interventions can have positive impact on the academic achievements and psychosocial development of adolescents. This paper describes the design of an effectiveness study of the online intervention “The Growth Factory” developed to empower adolescents with intellectual disabilities by teaching a growth mindset. In addition, with this study we will also contribute to a further understanding of possible moderating and mediating effects of mindset interventions. By doing so we gain more insight into what works for whom and how it works when it comes to interventions aiming to develop a growth mindset. Furthermore, this is the first study evaluating an online mindset intervention specifically adapted to adolescents with intellectual disabilities. If “The Growth Factory” turns out to be effective, a significant contribution will be made to the evidence-based treatment empowering adolescents with intellectual disabilities.

## Abbreviations

BPM-P: Brief Problem Monitor-Parent
BPM-Y: Brief Problem Monitor-Youth
EMPO Youth 2.0: Empowerment Youth 2.0
MQ: Mindset Questionnaire
MYTS: Motivation for Youth’s Treatment Scale
RCT: Randomized controlled trial
RSES: Rosenberg Self-Esteem Scale
SRS: Session Rating Scale
T0: Pre-test
T1: Post-test
T2: Follow-up at three months
T3: Follow-up at six months
TASC-r: Therapeutic Alliance Scale for Children, revised
